# What Should Primary Prevention in Burnout Look Like? Promoting Attributes, Roles and Social Networks with Instrumental Outcomes

**DOI:** 10.1007/s40670-024-02276-6

**Published:** 2025-01-10

**Authors:** Sean Naughton, Liliana Marques, Fergus Murphy, Mary Clarke

**Affiliations:** 1 School of Medicine, University College Dublin, Dublin, Ireland; 2 DETECT Early Intervention in Psychosis Service, Dublin, Ireland

**Keywords:** Burnout, Primary prevention, Medical students, Residents, Curiosity, Mentorship

## Abstract

Burnout remains a vexing issue for healthcare workers, educators and policy makers. Continuing high prevalence rates have focused the need for new and innovative approaches. The deleterious personal and professional consequences of burnout when it does develop place this focus on primary prevention. Yet despite its benefits, primary prevention initiatives have several barriers to engagement which impact their effectiveness. In this article, the authors explore potential barriers to engagement with primary level prevention of burnout amongst physicians-in-training, many of which are rooted in the social and professional contexts of training. Understanding the motivations of physicians-in-training should be used to guide the development of initiatives which combine protection against burnout with relevant goal-directed, instrumental outcomes. Three principles which can guide initiatives combining these features are explored. Promoting curiosity and curiosity-led enquiry combines a well-established protective attribute with educational and professional benefits. Curiosity-led endeavours can be developed into areas of special interest and competence, harnessing the protective benefits of self-efficacy and peer acknowledgement. Finally, special interests and the professional roles to which they lead foster the development of professional social networks and mentorship relationships. These are protective, particularly for physicians-in-training navigating role and organisational transitions. Supporting interventions with instrumental outcomes is both beneficial in engaging investment and also sustainable across the career-span. Burnout remains a challenging issue, and while programmatic interventions continue to have a role, the benefits of broader primary preventative approaches should be considered in terms of the potential engagement and sustainability advantages they confer.

**Supplementary Information:**

The online version contains supplementary material available at 10.1007/s40670-024-02276-6.

## Introduction

Burnout has been the focus of substantial investigative effort, the past decade seeing an almost exponential increase in studies profiling prevalence rates amongst healthcare workers [1]. This reflects the salience of a syndrome which concurrently impacts the health of workers, the quality of their work and the organisations which host them [2]. In an era of physician workforce shortages which are projected to increase, [3] burnout has become a matter of parallel personal, professional and national concern.

Five decades of enquiry have yielded insights. These include conceptual definitions, assessment schedules, risk factors and most broadly success in focusing attention on the consequences of caring work [4–6]. Yet prevalence rates remain high [7]. At the point where burnout results in sick leave, it is likely to be prolonged with a substantial portion (25–50%) not recovered after 2 to 4 years [8–10]. The recognition that the development of burnout is a slow process [4] has highlighted the need to focus on primary prevention strategies. However, this remains a key gap in the field [6].

Conceptualisations of the developmental pathway to burnout highlight potential junctures of intervention. The social cognitive theory of burnout places self-efficacy and self-confidence at the foot of its development [11, 12] whereby doubts held by professionals about their competence may precipitate a ‘self-efficacy crisis’ noted to prelude burnout [13]. Social exchange theory considers the need for workplace-based acknowledgement of efforts, in whose absence the inequity between effort and reward generates emotional exhaustion [14]. Other frameworks highlight organisational structures, demand-resource matching and maladaptive initial responses to overwhelm [15].

Although the most efficacious level of intervention, the successes of primary prevention initiatives are limited by engagement rates. This is mediated by factors including insufficient knowledge of downstream risks, uncertain relevance to the ‘at-risk’ individual and social messaging [16]. To tender a meaningful impact on prevalence rates, the question must broaden from the *efficacy* of primary prevention initiatives for burnout to their *effectiveness*, a concept which combines efficacy with tolerability and engagement under real-world conditions [17]. This paper examines  barriers to engagement in primary prevention initiatives for burnout amongst physicians-in-training. It proposes solutions relevant to training and across the career-span.

## Physicians-in-Training: Where Primary Prevention Begins

Selecting a target population is a key component of a primary prevention strategy. Care must be taken, as strategies overly fitted to certain populations can narrow their reach, sometimes termed the *prevention paradox* [18]. Physicians-in-training, which include medical students and residents, are an important group because they are not just a sub-population of the group at risk, but an obligatory stage through which all physicians must transit. Training is the earliest point at which individuals are inducted into the physician cohort, assuming its attendant elevated burnout risk. This must be where primary prevention begins.

Medical students enter undergraduate training with equal or lower rates of burnout than demographically matched peers [19] but as training progresses their markers of mental health diverge and deteriorate [20]. Stress, anxiety and burnout are higher amongst medical students compared to peers [21] with a consequent burnout prevalence of 44.2%, or almost half of all medical students [22]. Many elements of the learning environment correlate with burnout severity, including clerkship organisation, [23] inadequate training experiences [24] and stressful rotations [25]. Clinical training takes place during a life stage of personal upheaval, where training may mandate relocations away from established social support structures [26] limiting the ability of students and trainees to engage native support systems [27].

Yet the stress inherent with medical training is a complex issue which belies simple environmental redesign. At least a component of this stress is mediated by the need to constantly engage in new and unfamiliar environments which spurs professional growth. Studies exploring the association of motivation and learning with stress produce a familiar curvilinear relationship, often rendered as the Yerkes-Dodson law [28]. A core role of educators is to support trainees to move beyond their comfort zone into a zone of proximal development, by definition a zone of discomfort [29]. There is a need to maintain performance standards and deliver minimum skillsets, delivering on the obligation which training organisations have to stakeholders beyond students including regulators and broader society [30]. An appropriate response to student burnout has challenged medical educators, where the need to balance professional development with wellbeing has led to difficulties distinguishing necessary from unnecessary discomfort [31].

## Engagement with Primary Prevention: The Need to Consider Motivation

As a syndrome predominantly affecting healthcare workers [32] and with many similarities to mental illness [33], it is perhaps unsurprising that most evaluated prevention initiatives are programmatic in nature, predominantly modelled on psychotherapeutic approaches [1]. Such programmes include mindfulness, psychoeducation, exercise, and cognitive behavioural therapy-based interventions [34]. While organisationally focused preventative strategies are efficacious, they are rare and most commonly involve simple reductions in workload or schedule changes, which due to the financial and organisational implications limit their widespread adoption [35]. Sustainable strategies with a long-term focus are required.

Engagement in such a putative intervention is predicated on the recruitment of interest from physicians-in-training, who will implicitly evaluate its perceived relevance to them. When prevention programmes have been introduced, uptake can be low and attrition high, prompting the response that such programmes should be compulsory [36]. Students have described the time commitments of such programmes as an added stressor, which can result in protest [37]. When students identify their own preference for self-care activities, programmatic activities rate low [37].

Medical students are not primarily motivated to prevent burnout. Their entry into clinical training is driven by a complex array of motivations, including competitiveness, prestige and the building of personal social capital, in addition to prosocial drives [38]. Training programmes serve not only as a means of knowledge accumulation but also as a process of professional socialisation [39]. The separation from pre-existing identities and support systems renders trainees susceptible to adopting the values of their new environment, [40] often transmitted covertly through the ‘hidden curriculum’[41]. This can have a powerful inhibitory effect on help-seeking [42] and may challenge attempts to place programmatic burnout interventions within educational programmes.

Motivation to engage in prevention can also be antagonised by competing priorities such as external pressures and students’ own high standards for success [43]. Medical students have reflected the challenge of balancing investment in their wellness with intrinsically and extrinsically motivated demands and emphasise the need to consider the feasibility of proposed interventions [44].

This is the challenge which any primary prevention intervention must navigate—the investment of time and effort such programmes require may only be made by individuals once they begin to experience burnout. This is especially true for a group like physicians-in-training, already conscious of time constraints. Yet the challenge generated by these priorities could also be viewed as a strength—physicians-in-training are already a highly motivated group. Medical training is a long and difficult endeavour which involves consistently deploying deliberate behaviour to achieve a goal. Such behaviour has been termed *instrumental behaviour*, from the behaviourist tradition which explores how and why repeated actions are deployed to achieve a desired goal [45]. We should consider the potential within such behaviours. Goal-directed, instrumental behaviour resulting from the anticipated achievement future goals, or *instrumental outcomes*, is a potent source of motivation [46]. If we are to consider prevention strategies for burnout against the benchmark of effectiveness, we must align them with the existing motivations of physicians-in-training. By framing protective interventions in terms of their potential to achieve future goals, rather than merely avoiding future adversity, engagement could be significantly improved.

## Primary Prevention Strategies with Instrumental Outcomes

### Promoting Attributes: Curiosity

Scaling the learning curve of clinical training in the ambiguous and context-rich environment of medicine requires motivation and curiosity. Curiosity, a core component of intrinsic motivation [47], drives the acquisition of new knowledge and motivates exploratory behaviour [48]. While relatively underexplored in the medical education literature, curiosity has been a subject of interest in organisational psychology literature for decades [48]. Curiosity is disproportionately represented among professionals who rely on innovative thinking [49] as it fosters engagement with complex and ambiguous topics and has been central to several scientific breakthroughs [50]. Given its generative effects, several multinational corporate firms have defined as one of their key values [51].

The application of curiosity to burnout prevention is that it both drives the acquisition of new knowledge and also fosters resilience by enhancing an individual’s ability to cope with uncertainty. Curiosity is stimulated in complex and ambiguous professional and educational environments [52]. Intolerance of ambiguity is centrally implicated in the development of burnout in both learners [53] and healthcare professionals [54]. Curiosity enhances the ability to navigate uncertainty facilitating mastery and increasing one’s sense of competence [55]. It can be activated in response to unresolved work problems, where motivated exploration leads to mastery and an increase in one’s sense of competence [56].

Curiosity’s protective effect has been investigated outside of healthcare settings. The stress generated by novel situations is buffered by curiosity, increasing resilience [57]. It is a dynamic coping response which has a moderating effect in high-demand workplaces. This was demonstrated during the COVID-19 pandemic, where the burnout risk conferred by work intensification was moderated by curiosity [58]. The protective effect of curiosity in this context may be mediated by the effect it exerts in high-demand, high-stress contexts, whereby active coping is engaged by increasing sources of stimulation and reinforcement, while decreasing stressors [59].

Despite its potential benefits, curiosity has until recently been largely overlooked in clinical burnout research, despite calls for this application [60]. Educational application of curiosity within medical education settings have been explored. Supporting curiosity in these settings increases active engagement with a view to preparing students for the context-rich, ambiguous world of clinical medicine [61]. Yet we should not assume that engagement in medical education will passively generate curiosity. A study profiling curiosity in medical students found it unchanged across a 4-year undergraduate programme [62]. Curiosity needs to be supported actively. Interventions to enhance curiosity are effective, across a variety of intervention principles, enhancing life satisfaction, work engagement and academic performance [63]. Social environments also have a role, with social cues influencing individuals’ curiosity about scientific questions [64]. These provide the principles for medical educators to develop curricula to enhance curiosity. As a first step, incorporating direct measures of curiosity in the evaluation of training programmes would guide the implementation of new initiatives [65].

Promoting curiosity early both fosters intellectual growth and equips physicians-in-training with a vital coping mechanism to thrive in challenging environments and mitigate the risk of burnout, combining resilience with a valued instrumental outcome.

### Promoting Roles: Special Interests and Areas of Competence

Beyond the intrinsically protective effect of curiosity-led professional exploration, the result, developing an area of particular expertise and competence also confers protection against burnout. Feelings of professional self-efficacy are strongly protective against burnout [66]. Workplace demands may be interpreted as either potential opportunities for professional growth, or hinderances, and self-efficacy moderates this relationship [67]. This perception triggers differential coping responses with demands which are perceived positively being associated with improved performance, motivation, job satisfaction and attitudes toward work, and demands which are perceived negatively being associated with job searching and turnover intention [68].

For physicians-in-training, cultivating special skills which garner peer and organisational recognition fosters social acknowledgement and appreciation, leading to higher professional fulfilment and lower burnout rates [69]. The cultivation of special interests is an ideal mechanism to develop self-efficacy, leading to professional growth and recognition [66]. Pursuing non-clinical professional roles, particularly those which yield career benefits such as peer-reviewed publications or academic advancement, is inversely correlated with burnout and emotional exhaustion [70, 71].

There is a need for training organisations to understand barriers and support engagement in professional special interests, if this protective benefit is to be realised. Clinical research offers the most well-developed case-study. Even where medical students are interested in undertaking research, lack of sufficient time, access to mentorship and institutional recognition and incentives become barriers [72]. These barriers can be addressed, and indeed programmes to formalise access to erstwhile ad-hoc research networks are developing [73, 74]. Consistently implementing such programmes and generating equivalent support for areas of special interest beyond research are important next steps. Supporting curiosity to develop into areas of special interest and competence recognises that the achievement of instrumental outcomes and their pursuit are protective.

### Promoting Structures: Professional Social Networks

While avoiding burnout may not be the primary motivation of physicians-in-training, socially and professionally assimilating into a new professional role is [75]. Special interests allow physicians-in-training to assume professional roles, as emerging clinical, educational or management experts, in turn fostering the development of professional social networks. These networks are particularly important during transitions to new organisations and increasingly senior roles [76]. Facilitating the establishment of professional social networks should be a core focus of burnout interventions given their well-established protective effects [77, 78].

Previous attempts to leverage this benefit have primarily focused on network building through social activities, such as choir groups, supportive communities and facilitated social activities [79, 80]. However, physicians-in-training often report that educational obligations antagonise their ability to maintain informal networks, particularly when they transition to the clinical phase of training [81]. Professional networks established in the course of pursuing self-motivated investigation should be considered an important resource. This is especially true for physicians-in-training new to organisations. Individuals who engage in self-motivated discovery have been found to ask for more feedback, request more information when confronted with ambiguous situations and are more open to receiving information from others [82]. Consistently acting on these feelings serves to strengthen social relationships, [56] which enhances a sense of belonging in work and increased fulfilment [83].

Professional social networks have been explored in relation to supporting clinical decision-making [84] and learning [85]. Many training organisations are themselves professional networks, and yet membership of such organisations will not passively recruit their protective benefits, the type and nature of engagement modulates their impact [86]. The foundational unit of a professional network is a mentorship relationship, which have demonstrated benefits against burnout [87]. Training organisations can support their development through structured roles, such as clinical fellowships. These posts can be configured to achieve a number of instrumental outcomes, including developing subspeciality expertise, while in tandem recruiting mentors who can advise, support and facilitate role transition at a challenging time [76]. Mentors can both model the benefit of a professional network and ‘sponsor’ the entry of mentees into broader professional networks as they progress through training [76].

## Sustaining Protective Interventions Across the Career-Span

### Job-Crafting

Burnout is a career-long risk, and so any prevention strategy should be measured across this interval. This is a key advantage of pursuing preventative interventions with an instrumental outcome focus. Organisational support of these initiatives should include providing physicians with the reasonable autonomy to pursue curiosity-driven endeavours and special interests alongside core clinical roles. The autonomy for physicians to orientate themselves to the most meaningful aspects of their role is protective. Self-perceived meaningfulness of work is an important component of professional fulfilment [69]. Physicians who spend less than 20% of their work time on the activity that is the most meaningful to them have double the rates of burnout [88]. This ‘job crafting’ approach has a mutually beneficial relationship with work-engagement whereby the gains generated increase engagement in a positive loop [89]. Workers who are permitted to job craft do so in a way that enhances their ability to perform their core role, [90] further incentivising engagement with the process.

### Sabbaticals

A model with an extensive heritage in academia and some commercial organisations is the sabbatical. Sabbaticals have been an established practice in academia since the nineteenth century, originally introduced to allow academics to concentrate on areas of professional interest, to learn new techniques and to stimulate intellectual and professional renewal [91]. In commercial organisations, their purpose is not only burnout prevention, but also to imbue employees with increased knowledge and creativity [92]. Although predating the present-day conceptualisation of burnout, they implicitly addressed burnout at its root, offering a break from routine commitments to promote long-term engagement. This distinguishes such interventions from simply resting which would be unlikely to be either effective or organisationally sustainable. Sabbaticals are also effective strategies at managing burnout when it has occurred [93, 94].

While implementing similar practices in healthcare may need consideration given concerns about staffing and continuity of care, these barriers are not insurmountable. Mini-sabbaticals, careful planning, and targeted implementation can help mitigate these challenges, providing substantial long-term benefits [95]. Short-term costs should be considered in the context of the costly financial burden of physician shortages and job turnover. As a long-term investment, the cost–benefit analyses of sabbaticals are favourable [96].

## Conclusion

Burnout prevention is a major contemporaneous challenge within healthcare. Research to date has made important contributions to elucidating the role of stress and job overload in the development of burnout. It is crucially important that organisational efforts continue to support physicians to maintain balance with life outside of work and avoid overload within it. However, given that burnout rates in physicians continue to be high, complementary strategies are required. Stress reduction alone does not equate to professional success. Individuals who remain in a field but disengaged may experience little stress but also little sense of professional fulfilment. While work-overload is harmful, the lack of meaningful roles and responsibilities is similarly harmful. Indeed, burnout itself is characterised by the tendency to conserve only ‘essential’ activities [4].

This paper proposes both a reconsideration of how primary prevention for burnout is considered and the benchmarks against which it is measured. Many of the principles proposed are the subject of existing academic attention outside of burnout, however by reframing their benefit in this regard support for their implementation and further research can be recruited.

Further research is needed to explore how educational and professional development programmes incorporating these principles perform against benchmarks of efficacy, feasibility, acceptability and ultimately effectiveness in preventing burnout. Mediating factors and individual-level differences will add further to the picture, refining implementation.

Middle life has been conceptualised as being a tension between generation and stagnation [97] with most physicians motivated to pursue their field by the former. By considering the goals and motivations of physicians from the earliest point in their training, we can provide them with skills and roles which they engage consistently rather than assuming they will only be deployed once burnout has set in. Adopting approaches that emphasise the effectiveness of burnout prevention interventions addresses this issue at a larger scale, benefiting both physicians and the patients they serve.

## Supplementary Information

Below is the link to the electronic supplementary material.

Supplementary file1 (DOCX 37 KB)
