# Supplementary material for: What Should Primary Prevention in Burnout Look Like? Promoting Attributes, Roles and Social Networks with Instrumental Outcomes
Source: Med Sci Educ. 2025 Jan 10;35(2):1093–100. doi: 10.1007/s40670-024-02276-6 (PMC12058567; doi:10.1007/s40670-024-02276-6)
Supplement: Supplementary file 1 — Supplementary file1 (DOCX 37 KB) [file 40670_2024_2276_MOESM1_ESM.docx]

| **Supplemental Table 1. Table of References by Topic** | | | | |
| --- | --- | --- | --- | --- |
| **No.** | **Year** | **Authors** | **Title** | **Journal** |
| **Burnout in healthcare populations** | | | | |
| 1 | 2020 | S De Hert | Burnout in healthcare Workers: Prevalence, impact and preventative strategies | Local and Regional Anesthesia |
| 2 | 2019 | National Academies of Sciences and Medicine | Taking action against clinician burnout: A systems approach to professional well-being. | N/A |
| 3 | 2020 | X Zhang et al. | Physician workforce in the United States of America: forecasting nationwide shortages | Human Resources for Health |
| 4 | 2001 | C Maslach et al. | Job burnout | Annual Review of Psychology |
| 5 | 2009 | WB Schaufeli et al. | Burnout: 35 years of research and practice | Career Development International |
| 6 | 2021 | E Demerouti et al. | New directions in burnout research | European Journal of Work and Organizational Psychology |
| 7 | 2019 | ZX Low et al. | Prevalence of burnout in medical and surgical residents: A meta-analysis | International Journal of Environmental Research and Public Health |
| 8 | 2021 | VL Dalgaard et al. | Cognitive impairments and recovery in patients with work-related stress complaints – four years later | Stress |
| 9 | 2016 | A Eskildsen et al. | Cognitive impairments in former patients with work-related stress complaints – one year later | Stress |
| 10 | 2012 | A van Dam et al. | Impaired cognitive performance and responsiveness to reward in burnout patients: Two years later | Work & Stress |
| **Developmental models of burnout** | | | | |
| 11 | 2002 | AM Pines | Teacher burnout: A psychodynamic existential perspective | Teachers and Teaching |
| 12 | 1993 | C Cherniss et al. | Professional burnout: Recent developments in theory and research | Taylor & Francis |
| 13 | 2005 | S Llorens et al. | Burnout como consecuencia de una crisis de eficacia: un estudio longitudinal en profesores de secundaria | Revista de Psicología del Trabajo y de las Organizaciones |
| 14 | 2011 | WB Schaufeli et al. | Stability and change in burnout: A 10‐year follow‐up study among primary care physicians | Journal of Occupational and Organizational Psychology |
| 15 | 2022 | S Edú-Valsania et al. | Burnout: A review of theory and measurement | International Journal of Environmental Research and Public Health |
| **Primary prevention** | | | | |
| 16 | 2015 | P Moreno-Peral et al. | Primary care patients' perspectives of barriers and enablers of primary prevention and health promotion-a meta-ethnographic synthesis | PLoS One |
| 17 | 2011 | HJ Möller | Effectiveness studies: advantages and disadvantages | Dialogues in Clinical Neuroscience |
| 18 | 2018 | C Thompson | Rose's prevention paradox | Journal of Applied Philosophy |
| **Burnout in medical students** | | | | |
| 19 | 2014 | CM Brazeau et al. | Distress among matriculating medical students relative to the general population | Academic Medicine |
| 20 | 2016 | L Dyrbye et al. | A narrative review on burnout experienced by medical students and residents | Medical Education |
| 21 | 2006 | LN Dyrbye et al. | Systematic review of depression, anxiety, and other indicators of psychological distress among U.S. and Canadian medical students | Academic Medicine |
| 22 | 2019 | A Frajerman et al. | Burnout in medical students before residency: A systematic review and meta-analysis | European Psychiatry |
| 23 | 2009 | LN Dyrbye et al. | The learning environment and medical student burnout: a multicentre study | Medical Education |
| 24 | 2018 | FJ Attenello et al. | Factors associated with burnout among US neurosurgery residents: a nationwide survey | Journal of Neurosurgery |
| 25 | 2018 | TM Jenkins et al. | Stressing the journey: using life stories to study medical student wellbeing | Advances in Health Sciences Education |
| 26 | 2021 | AB Parikh | On the transition to attendinghood | Journal of Cancer Education |
| 27 | 1996 | J Koniarek et al. | Social support as a buffer in the stress-burnout relationship | International Journal of Stress Management |
| **Stress and performance** | | | | |
| 28 | 1994 | KH Teigen | Yerkes-Dodson: A law for all seasons | Theory & Psychology |
| 29 | 2022 | AK Kumagai | Discomfort, doubt, and the edge of learning | Academic Medicine |
| 30 | 2011 | S Lindgren et al. | Social accountability of medical education: aspects on global accreditation | Medical Teacher |
| 31 | 2024 | L Rosenbaum | Being well while doing well - Distinguishing necessary from unnecessary discomfort in training | New England Journal of Medicine |
| **Current interventions for primary prevention in burnout** | | | | |
| 32 | 2012 | TD Shanafelt et al. | Burnout and satisfaction with work-life balance among US physicians relative to the general US population | Archives of Internal Medicine |
| 33 | 2016 | C Maslach et al. | Understanding the burnout experience: recent research and its implications for psychiatry | World Psychiatry |
| 34 | 2023 | DJ Madigan et al. | Interventions to reduce burnout in students: A systematic review and meta-analysis | European Journal of Psychology of Education |
| 35 | 2017 | M Panagioti et al. | Controlled interventions to reduce burnout in physicians: A systematic review and meta-analysis | JAMA Internal Medicine |
| **The implementation of current primary prevention strategies** | | | | |
| 36 | 2021 | R Waechter et al. | Mitigating medical student stress and anxiety: Should schools mandate participation in wellness intervention programs? | Medical Teacher |
| 37 | 2017 | EE Ayala et al. | What do medical students do for self-care? A student-centered approach to well-being | Teaching and Learning in Medicine |
| **The social context of medical training** | | | | |
| 38 | 2019 | C Olsson et al. | Sociological analysis of the medical field: using Bourdieu to understand the processes preceding medical doctors' specialty choice and the influence of perceived status and other forms of symbolic capital on their choices | Advances in Health Sciences Education |
| 39 | 2015 | B Vaidyanathan | Professional socialization in medicine | AMA Journal of Ethics |
| 40 | 1961 | HS Becker | Boys in white: Student culture in medical school | N/A |
| 41 | 2018 | C Lawrence et al. | The hidden curricula of medical education: A scoping review | Academic Medicine |
| 42 | 2015 | LN Dyrbye et al. | The impact of stigma and personal experiences on the help-seeking behaviors of medical students with burnout | Academic Medicine |
| 43 | 2011 | RF Kushner et al. | Using behavior change plans to improve medical student self-care | Academic Medicine |
| 44 | 2024 | M Prendergast et al. | Burnout in early year medical students: experiences, drivers and the perceived value of a reflection-based intervention | BMC Medical Education |
| **Instrumental behaviours and outcomes** | | | | |
| 45 | 2002 | RE Clarke | Classical conditioning | Encyclopedia of the Human Brain |
| 46 | 2016 | LK Fryer et al. | Understanding students' instrumental goals, motivation deficits and achievement: Through the lens of a latent profile analysis | Psychologica Belgica |
| **Curiosity: conceptual background** | | | | |
| 47 | 1992 | U Schiefele et al. | Interest as a predictor of academic achievement: A meta-analysis of research | N/A |
| 48 | 1994 | G Loewenstein | The psychology of curioisty: A review and reinterpretation | Psychological Bulletin |
| 49 | 2016 | P Celik et al. | Work-related curiosity positively predicts worker innovation | Journal of Management Development |
| 50 | 2009 | N Rosenberg | Some critical episodes in the progress of medical innovation: An Angle-American perspective | Research Policy |
| 51 | 2022 | F Lievens et al. | Killing the cat? A review of curiosity at work | Academy of Management Annals |
| **Curiosity: protective effects** | | | | |
| 52 | 1994 | M Zuckerman | Impulsive unsocialized sensation seeking: The biological foundations of a basic dimension of personality | Temperament: Individual differences at the interface of biology and behavior. |
| 53 | 2022 | J Xu et al. | Coping with students’ stress and burnout: Learners’ ambiguity of tolerance | Frontiers in Psychology |
| 54 | 2009 | T Tunc et al. | Role conflict, role ambiguity, and burnout in nurses and physicians at a university hospital in Turkey | Nursing & Health Scienecs |
| 55 | 1996 | KE Weick | Prepare your organization to fight fires | Harvard Business Review |
| 56 | 2020 | TB Kashdan et al. | Curiosity has comprehensive benefits in the workplace: Developing and validating a multidimensional workplace curiosity scale in United States and German employees | Personality and Individual Differences |
| **Curiosity in the workplace** | | | | |
| 57 | 2000 | ED Pulakos et al. | Adaptability in the workplace: Development of a taxonomy of adaptive performance | Journal of Applied Psychology |
| 58 | 2023 | LM Blanco-Donoso et al. | Work intensification and its effects on mental health: The role of workplace curiosity | The Journal of Psychology |
| 59 | 2017 | E Garrosa et al. | How do curiosity, meaning in life, and search for meaning predict college students’ daily emotional exhaustion and engagement? | Journal of Happiness Studies |
| **Curiosity in medical training** | | | | |
| 60 | 2019 | A Schattner | An antidote to burnout? Developing broad-spectrum curiosity as a prevailing attitude | QJM: An International Journal of Medicine |
| 61 | 2011 | L Dyche et al. | Curiosity and medical education | Medical Educator |
| 62 | 2017 | R Sternszus et al. | Describing medical student curiosity across a four year curriculum: An exploratory study | Medical Teacher |
| 63 | 2023 | NS Schutte et al. | A meta-analytic investigation of the impact of curiosity-enhancing interventions | Current Psychology |
| 64 | 2021 | R Dubey et al. | Curiosity Is contagious: A social influence intervention to induce curiosity | Cognitive Science |
| 65 | 2022 | AM Sullivan et al. | The Harvard Medical School Pathways curriculum: A comprehensive curricular evaluation | Medical Teacher |
| **Self-efficacy and burnout** | | | | |
| 66 | 1989 | C Cherniss | Burnout in new professionals: A long-term follow-up study | Journal of Health and Human Resources Administration |
| 67 | 1984 | RS Lazarus et al. | Stress, appraisal, and coping |  |
| 68 | 2015 | M Ventura et al. | Professional self-efficacy as a predictor of burnout and engagement: the role of challenge and hindrance demands | The Journal of Psychology |
| 69 | 2022 | DW Lu et al. | Drivers of professional fulfillment and burnout among emergency medicine faculty: A national wellness survey by the Society for Academic Emergency Medicine | Academic Emergency Medicine |
| 70 | 2009 | TD Shanafelt et al. | Burnout and career satisfaction among American surgeons | Annals of Surgery |
| 71 | 1994 | S Lloyd et al. | Burnout, depression, life and job satisfaction among Canadian emergency physicians | Journal of Emergency Medicine |
| **Interventions to promote self-efficacy** | | | | |
| 72 | 2024 | B Mokhtari et al. | The next generation of physician-researchers: undergraduate medical students' and residents' attitudes, challenges, and approaches towards addressing them | BMC Medical Education |
| 73 | 2012 | K Zier et al. | An innovative portfolio of research training programs for medical students | Immunologic Research |
| 74 | 2007 | C O'Connor Grochowski et al. | A curricular model for the training of physician scientists: the evolution of the Duke University School of Medicine curriculum | Academic Medicine |
| **Professional social networks and burnout** | | | | |
| 75 | 1965 | SW Bloom | The sociology of medical education: Some comments on the state of a field | The Milbank Memorial Fund Quarterly |
| 76 | 2023 | S Naughton et al. | Post-CSCST fellowships: beyond subspecialization | Irish Journal of Medical Science |
| 77 | 2021 | E Sesemann et al. | Healthcare employees' social networks, burnout, and health | Families, Systems & Health |
| 78 | 2016 | E Rogers et al. | Getting by with a little help from friends and colleagues: Testing how residents' social support networks affect loneliness and burnout | Canadian Family Physician |
| 79 | 2023 | C Cohen et al. | Workplace interventions to improve well-being and reduce burnout for nurses, physicians and allied healthcare professionals: a systematic review | BMJ Open |
| 80 | 2022 | AM Aghaei et al. | A social network intervention to improve connectivity and burnout among psychiatry residents in an academic institution: a quasi-experimental study | BMC Medical Education |
| 81 | 2017 | L Dunham et al. | Medical student perceptions of the learning environment in medical school change as students transition to clinical training in undergraduate medical school | Teaching and Learning in Medicine |
| 82 | 2017 | SH Harrison et al. | Pliable guidance: A multilevel model of curiosity, feedback seeking, and feedback giving in creative work | Academy of Management Journal |
| 83 | 2007 | S Thau et al. | Self-defeating behaviors in organizations: The relationship between thwarted belonging and interpersonal work behaviors | Journal of Applied Psychology |
| **Interventions to support professional social networks** | | | | |
| 84 | 2013 | DA Cohen et al. | The influence of a professional physician network on clinical decision making | Patient Education and Counseling |
| 85 | 2021 | M Levy et al. | The decision-making and learning roles of a professional social network: The case of a family physicians’ network | International Journal of Medical Informatics |
| 86 | 2012 | FC Cunninghamet al. | Health professional networks as a vector for improving healthcare quality and safety: a systematic review | BMJ Quality & Safety |
| 87 | 2022 | K Cavanaugh et al. | The positive impact of mentoring on burnout: Organizational research and best practices | Journal of Interprofessional Education & Practice |
| **The protective effects of job crafting** | | | | |
| 88 | 2009 | TD Shanafelt et al. | Career fit and burnout among academic faculty | Archives of Internal Medicine |
| 89 | 2017 | AB Bakker et al. | Job demands-resources theory: Taking stock and looking forward | Journal of Occupational Health Psychology |
| 90 | 2020 | G Jutengren et al. | The potential importance of social capital and job crafting for work engagement and job satisfaction among health-care employees | International Journal of Environmental Research and Public Health |
| **Sabbaticals to sustain protective effects across the career-span** | | | | |
| 91 | 1989 | JB Reuler | Sabbatical | JAMA |
| 92 | 2005 | AE Carr et al. | Sabbaticals and employee motivation: Benefits, concerns, and implications | Journal of Education for Business |
| 93 | 2010 | C Kang et al. | The effects of a month-long sabbatical program on helping professionals of nonprofit human service organizations in South Korea: Burnout, general Health, organizational commitment, and the sense of well-being | Administration in Social Work |
| 94 | 2010 | OB Davidson et al. | Sabbatical leave: who gains and how much? | Journal of Applied Psychology |
| 95 | 2020 | JG Leung et al. | The professional sabbatical: A systematic review and considerations for the health-system pharmacist | Research in Social and Administrative Pharmacy |
| 96 | 2012 | GL Schaar et al. | Nursing sabbatical in the acute care hospital setting: a cost-benefit analysis | The Journal of Nursing Administration |
| 97 | 2016 | JC Malone et al. | Midlife Eriksonian psychosocial development: Setting the stage for late-life cognitive and emotional health | Developmental Psychology |
